# Development of the Social and Emotional Competence Assessment Battery for Adults

**DOI:** 10.1177/10731911221127922

**Published:** 2022-09-30

**Authors:** Sofia Oliveira, Magda Sofia Roberto, Ana Margarida Veiga-Simão, Alexandra Marques-Pinto

**Affiliations:** 1 Universidade de Lisboa, Faculdade de Psicologia, Centro de Investigação em Ciência Psicológica, Lisboa, Portugal

**Keywords:** adults, assessment, confirmatory factor analysis, psychometric study, reliability, social and emotional competence, validity

## Abstract

Literature has emphasized the urgency of investing in the promotion of Social and Emotional Competence (SEC) in adults. Therefore, the development of a theoretically grounded and developmentally adjusted measure that adequately assesses SEC in its different domains is needed. This study aimed to develop a self-report battery for the assessment of adults’ SEC. Factor structure, reliability, and validity of the Social and Emotional Competence Assessment Battery for Adults (SECAB-A) were assessed. Seven-hundred and ninety-six adults (80.7% female) completed the SECAB-A. A subsample of 63 elementary school teachers (92.1% female) participated at two points in time and also completed external measures assessing affect, emotion regulation, and burnout symptoms, thus making it possible to test for test–retest reliability and convergent and discriminant validities. Despite sample size variation, no statistically significant differences between groups were found for the SECAB-A scales. Extraction of factors and confirmatory factor analysis supported the hypothesized factorial structures. Coefficient omegas suggested adequate internal consistency and scores were highly correlated between the two data collection waves, also ensuring adequate test–retest reliability. There was also evidence of the SECAB-A’s convergent and discriminant validities against the external measures. Results from this study indicate that the SECAB-A is a promising measure for the adult population. Nevertheless, additional criterion-related and construct validity research is needed.

Over the past 25 years, Social and Emotional Learning (SEL) has become a central interest, particularly within educational contexts (Durlak et al., 2015). SEL seeks to enhance individuals’ ability to effectively respond to the daily tasks and challenges of the twenty-first century, through the integration of cognitive, affective and behavioral skills (Durlak et al., 2015; Tolan et al., 2016). Accordingly, SEL is defined as the process through which the five core areas of Social and Emotional Competence (SEC), namely self-awareness, self-regulation, social awareness, relationship skills, and responsible decision making, are acquired. Even though the first wave of SEL interventions has targeted children and youths, the promising impacts found as a result of SEC development in children and youths (e.g., citizen contribution, work productivity, critical thinking, predisposition to lifelong learning, practice of safe and healthy behaviors, mental health and well-being; Durlak et al., 2011; Taylor et al., 2017), along with the changes and demands resulting from the globalization of work, have made SEC appealing to a broader target population of adults, namely college students and teachers (Durlak et al., 2015). Indeed, SEL interventions targeting teachers’ own SEC have gained momentum in recent years. Evidence has been found that these interventions contribute to increasing teachers’ job performance (Jennings et al., 2013, 1017) by promoting not only teachers’ SEC but also improving their well-being and preventing their psychological distress by reducing emotional exhaustion and increasing personal accomplishment (Oliveira et al., 2021a, 2021b). Additionally, motivated by the rapid changes currently faced by workplaces, accompanied by the guidelines of new school curricula, human resource practitioners in other fields such as business, leadership, and health care are making efforts to select and train a more social and emotionally competent workforce (Mattingly & Kraiger, 2019; Monnier, 2015). In line with the findings regarding the impacts of SEL interventions for teachers, interventions targeting these other professional groups also appear to contribute to workers’ health and performance (Martins et al., 2010; Mattingly & Kraiger, 2019). However, this human resource management line of research and practice has been guided by the Emotional intelligence theory which, despite being central to the SEL framework does not cover all five core areas of SEC, thus promoting unclear overlaps and distinctions (Oliveira et al., 2021a; Tolan et al., 2016). The SEL framework is much broader since it includes not only the intrapersonal skills of emotion recognition and emotional regulation, but also integrates and explicitly trains other cognitive and behavioral dimensions such as self-regulation, motivation regulation, self-efficacy, problem solving and decision making (Durlak et al., 2015; Tolan et al., 2016).

Moreover, despite the perceived positive impacts and importance of SEC promotion among adults, this is still a recent line of intervention and, consequently, research. The assessment of SEC is largely heterogeneous and still lacks consistency as different outcome variables, for instance, Emotional reappraisal, Understanding emotion, Emotional valence, Emotional regulation, Compassion, and multiple instruments, are used across the studies (Oliveira, Roberto, Pereira, et al., 2021). Furthermore, due to their origin, the initial aim of SEL interventions for teachers was to help them increase their ability to manage classrooms and teach their students (Greenberg et al., 2003). This approach led to an emphasis on the development of teachers’ interpersonal SEC (i.e., social awareness and relationship skills), which were then assessed mainly through their distal impacts, either at a professional level through observational measures of classroom practices or at a personal level, through the assessment of variables such as well-being, stress, and burnout (Oliveira, Roberto, Pereira, et al., 2021). When the studies assessed the direct, proximal impact of SEL interventions on SEC themselves, they tended to use instruments that evaluated specific skills within the scope of SEC (e.g., Emotional reappraisal as a representation of Self-regulation competence) and evaluated mainly intrapersonal-level SEC (i.e., self-awareness and self-regulation; Oliveira, Roberto, Pereira, et al., 2021). The same limitation is encountered in the resource management line of research, where multiple and overlapping operationalizations and several distinct measures are used without consensus (Mattingly & Kraiger, 2019). Nevertheless, in view of the promising potential of a more social and emotionally competent workforce for workers’ occupational health and well-being, performance and leadership, and interpersonal relations (Jennings et al., 2013, 2017; Mattingly & Kraiger, 2019; Monnier, 2015; Oliveira, Roberto, Pereira, et al., 2021; Oliveira, Roberto, Veiga-Simão, & Marques-Pinto, 2021), the interest in the promotion of SEC in adults across the lifespan will continue to increase. Thus, the development and validation of a theoretically grounded instrument to directly assess all the specific skills across the five core SEC in adults are sorely needed. Therefore, in an effort to contribute to the clarification and integration of concepts and promote a parsimonious and thorough assessment measure that contributes to filling the gap of practical and research needs across different fields (e.g., education, business, health), this study aims to develop a self-report battery for the assessment of social and emotional competencies in adults and to examine its factor structure, reliability, and validity.

## The SEL Framework

The SEL rationale emerged seeking to establish common ground for educational interventions that were increasing in schools at the time to prepare children and youths for the life challenges of the 21st century (Oliveira, Roberto, Pereira, et al., 2021). By the end of the 1990s, schools were overloaded with apparently nonrelated interventions targeting children and youths’ social, emotional, and behavioral skills. In this scenario, the SEL rationale was developed as a framework that aimed to operationalize the constructs underlying the upcoming interventions and define practical guidelines to inform and unify the design and evaluation of interventions aiming to promote those social, emotional, and behavioral skills—later defined as SEC (for an extensive review of the SEL rationale history see Oliveira, Roberto, Pereira, et al., 2021). Although the SEL rationale and interventions have primarily followed a practice-centered approach, theories on emotional intelligence, social skills training, and learning theories, among others, have been highlighted as those that should be guiding SEL interventions and research (Durlak et al., 2015; Osher et al., 2016; Tolan et al., 2016), informing on the specific contents to be addressed, the specific change-promoting strategies, and the specific target contexts/populations (Oliveira, Roberto, Pereira, et al., 2021). Indeed, these theories assisted in the trimming of the five core SEC domains and the specific skills within, which should be considered in the SEL rationale and interventions (Oliveira, Roberto, Pereira, et al., 2021). More specifically, the Emotional intelligence theory has offered a major contribution to the development of the more emotional and intrapersonal area of SEL, while the Social skills training framework has assisted the construction of the more social/interpersonal dimension (Marques-Pinto & Raimundo, 2016).

Therefore, with regard to the intrapersonal dimension of SEC, SEL is the process through which people develop their self-awareness (i.e., the ability to understand their own emotions, goals and values, and to have accurate self-perception and self-efficacy beliefs) and self-regulation (i.e., the ability to regulate their emotions and behaviors, namely through delayed gratification, stress management, goal setting and achieving, adaptability and/or impulse control; Durlak et al., 2015). In addition, concerning the interpersonal dimension of SEC, SEL should consider the promotion of people’s Social awareness (i.e., the ability to recognize others’ emotions, take perspective, empathize and feel compassion, respect others and appreciate diversity, and to understand social norms) and Relationship skills (i.e., the ability to establish and maintain healthy relationships and to act in accordance with social norms, namely, through clear communication, active listening, cooperation, conflict management, and seeking help; Durlak et al., 2015). Finally, SEL should promote the development of one’s Responsible decision-making (i.e., the ability to make ethical and constructive choices on personal behavior and social interactions across settings, to evaluate and reflect on personal behavior, and to effectively solve problems; Durlak et al., 2015). Despite the organization of SEC into these five main domains, where some represent a knowledge dimension (i.e., self-awareness and social awareness) while others depict a behavioral dimension (i.e., self-regulation and relationship skills), all these competencies are considered interrelated areas of knowledge and behavior (Durlak et al., 2015; Elias et al., 1997).

## Review of Existing Measures to Assess Adults’ SEC

Due to the SEL rationale history, robust empirical research and the development of validated psychometric measures are still needed to reduce between-studies heterogeneity and increase reliable empirical evidence of SEL contributions (Jennings et al., 2017; Monnier, 2015; Oliveira, Roberto, Pereira, et al., 2021). In fact, despite growing interest in teachers’ SEL and the recommendations for investment in the development of SEC among other adults and across fields of research (Durlak et al., 2015; Schonert-Reichl, 2017), to date, and to the best of our knowledge, no validated psychometric measure is available to assess the full range of adults’ specific skills across the five core SEC in accordance with the SEL rationale proposed by Elias et al. (1997) and Durlak et al. (2015).

When reviewing the existing measures, the available questionnaires were found to measure mainly intrapersonal skills, such as the *Emotional Regulation Questionnaire* (Gross & John, 2003), the *Positive and Negative Affect Schedule* (Watson et al., 1988), the *Trait Meta-Mood Scale* (TMMS; Salovey et al., 1995), and the *Emotional Skills and Competence Questionnaire* (ESCQ; Takšić et al., 2009). However, they were not developed in accordance with the SEL rationale, and each one assesses different specific skills within the intrapersonal domains of SEC (e.g., emotional regulation and emotional experience). Therefore, when considered individually, these instruments do not fully assess the intrapersonal aspects of SEC. Likewise, the measures found to assess the interpersonal skills (e.g., *Interpersonal Competence Questionnaire* [ICQ], Buhrmester et al., 1988; *Interpersonal Reactivity Index*, Davis, 1983) are not aligned with this framework as they do not cover all the features of the interpersonal SEC dimensions. Moreover, as far as interpersonal skills are concerned, the measures were found to be scarcer and more clinically focused (e.g., on personal discomfort in relationships or communication styles).

More global measures that include both intrapersonal and interpersonal competencies were found, such as the *Emotional Intelligence View 360* (Nowack, 2004), the *Emotional Quotient Inventory* (EQ-i; Bar-On, 1997), the *Feedback-Inventar zur berufsbezogenen Erstorientierung für Lehramtsstudierende* (Böttcher et al., 2007), and the *Questionari de Desarrollo Emocional para Adultos* (QDE-A; Pérez-Escoda et al., 2010). Nevertheless, once again these measures were not developed according to the SEL rationale and, although some of them were found to have followed the Emotional intelligence theory, none of them cover all the SEC domains demanding, once again, the use of multiple instruments for a complete assessment of these skills. Furthermore, some of these measures present psychometric validity issues and others were developed to measure not only SEC but also other related dimensions (e.g., independence or happiness). However, some intervention guides are available in the literature, namely, the *Self-Assessing Social and Emotional Instruction and Competencies: A Tool for Teachers* (SSEIC) by the American Institutes for Research (Yoder, 2014) and the *TOOL: Personal Assessment and Reflection—SEL Competencies for School Leaders, Staff, and Adults* by the Collaborative for Academic, Social, and Emotional Learning consortium (CASEL, 2019) which, despite not being aimed at psychometric assessment, offer prompts to develop items in terms of the strategies to promote growth across the five-core SEC.

Another aspect that calls for further reflection is the assessment of the interpersonal dimension of SEC. When assessing and describing the interpersonal dimension of SEC, both the preexistent measures and the intervention guides operationalize the items and indicators based on a behavioral expression of the skills being assessed even with regard to the social awareness domain (e.g., *empathy*, which is a specific skill within social awareness, is operationalized as “I listen actively and can grasp another person’s perspective and feelings from both verbal and nonverbal cues”; the same for *difference appreciation* which is measured through the indicator “I appreciate and get along with people of diverse backgrounds and cultures in my school community and utilize inclusionary practices to ensure all voices are represented”; CASEL, 2019). In fact, as interpersonal competencies are expressed through relations with others (Durlak et al., 2015), the knowledge and behavioral aspects of these domains cannot be fully separated (e.g., one can only be empathetic when showing empathy). Likewise, within the SEL framework, the interpersonal domains of SEC are often referred to in terms of the ability to build and maintain strong and supportive relationships and interact effectively with others on one hand, and the ability to effectively manage conflict situations and negative social interactions (e.g., Durlak et al., 2015; Elias et al., 1997; Jennings & Greenberg, 2009) on the other. In both these domains, the knowledge and behavioral aspects of the interpersonal SEC (in a more theoretical organization) are present. This is an important factor to bear in mind when assessing SEC and developing SEL interventions.

## Development of the Social and Emotional Competence Assessment Battery for Adults

The present study sought to develop the *Social and Emotional Competence Assessment Battery for Adults* (SECAB-A), a measure that proposes to assess the different specific skills within the five core domains of SEC in accordance with the SEL rationale. This battery was developed through the creation of three independent questionnaires: the *Intrapersonal Competence Questionnaire*, the *Interpersonal Competence Questionnaire*, and the *Responsible Decision-Making Competence Questionnaire*. Following the SEL rationale, having reviewed the existing measures and identified gaps, and aiming to cover the different specific skills across SEC, the following hypotheses were established with regard to the SECAB-A expected factor-structure:

**Hypothesis 1a (H1a):** The *Intrapersonal Competence Questionnaire* will present a two-factor solution with one scale assigned to a knowledge dimension (i.e., self-awareness) and one scale referring to a behavioral dimension (i.e., self-regulation).
**Hypothesis 1b (H1b):** The *Interpersonal Competence Questionnaire* will present a two-factor solution with one scale assigned to the establishment and maintenance of strong and supportive interpersonal relationships (i.e., positive relationship) and one scale referring to the ability to negotiate solutions and manage conflicts (i.e., conflict management).
**Hypothesis 1c (H1c):** The *Responsible Decision-Making Competence Questionnaire* will present a one-factor solution regarding responsible decision-making.

In addition, with a view to assessing the convergent and discriminant validity of the SECAB-A, some additional measures were used. To test the convergent validity of the *Intrapersonal competence questionnaire*, the *Cognitive reappraisal* scale of the *Emotional Regulation Questionnaire* (Gross & John, 2003) and the *Positive affect* scale of the *Positive and Negative Affect Schedule* (Watson et al., 1988) were used. These scales were chosen as they measure constructs that are expected to be related to both Self-regulation and Self-awareness, namely, being able to change the emotional impact of a situation by changing its meaning (Gross & John, 2003) and feeling positive affect on a daily basis (Watson et al., 1988). As no valid and robust measures were found to test convergent validity for both the interpersonal skills and the responsible decision-making competencies of Portuguese adults, convergent validity for the *Interpersonal competence questionnaire* and *Responsible decision-making questionnaire* was not assessed. Thus, the following hypothesis was established with regard to convergent validity:

**Hypothesis 2 (H2):** Moderate and positive intercorrelations between intrapersonal skills and cognitive reappraisal and positive affect are expected.

Concerning discriminant validity, symptoms of burnout were considered for the study. As previously reflected upon, due to their school-based origin, teachers represent a professional group in which SEL proves relevant and for which SEL impacts have been most studied across adult populations (e.g., Durlak et al., 2015). The literature has supported that most teacher-specific stressors are social and emotional related (McCarthy et al., 2016; Roeser et al., 2013). Therefore, as previously mentioned, SEC has been referred to as a buffer of psychological distress and burnout symptoms for teachers (Jennings et al., 2017; Oliveira, Roberto, Pereira, et al., 2021; Oliveira, Roberto, Veiga-Simão, & Marques-Pinto, 2021). Although research on SEL intervention impacts is still very heterogeneous, within this field of research, burnout, more specifically emotional exhaustion symptoms, is the most consistently studied variable across SEL interventions for teachers’ literature (Oliveira, Roberto, Pereira, et al., 2021; Oliveira, Roberto, Veiga-Simão, & Marques-Pinto, 2021) Therefore, to test discriminant validity, the two core symptoms of burnout (i.e., emotional exhaustion and depersonalization; Bresó et al., 2007) were used. Indeed, as *Emotional exhaustion* is a more internalized dimension referring to feelings of emotional distress (Maslach & Leiter, 2016), this dimension was expected to be discriminated from interpersonal skills and responsible decision-making. In the same vein, as *Depersonalization* is a more externalized symptom that is manifested through negative and detached behaviors toward others (Maslach & Leiter, 2016), this dimension was expected to be discriminated from intrapersonal skills. Hence, the following hypotheses were established with regard to discriminant validity:

**Hypothesis 3a (H3a):** Small, negative, and nonsignificant intercorrelations between interpersonal skills and responsible decision-making and emotional exhaustion are expected.
**Hypothesis 3b (H3b):** Small, negative, and nonsignificant intercorrelations between intrapersonal skills and depersonalization are expected.

## Method

### Participants

A sample of 796 Portuguese adults (80.7% female, *M* = 35.70 years, standard deviation [*SD*] = 12.84, range: 18–84 years) was considered in the study for data diagnostics and hypothesis testing regarding the SECAB-A expected factor structure. The majority of the participants had a bachelor’s (38.5%) or a master’s degree (36.7%). The set of participants included residents from all Portuguese countries to ensure a national representation. To enroll in the study, participants were required to be Portuguese native speakers, have Portuguese nationality, and to be aged 18 years or older.

In addition, to perform the test–retest and validity analyses, a convenience subsample of 63 elementary school teachers (92.1% female, *M* = 46.97 years, *SD* = 5.31, range: 37–60) was used. The majority of the participants had a master’s degree (69.8%) and were residents of the Lisbon Metropolitan Area. This convenience subsample was selected as it represents a professional group in which SEL proves relevant. It is also a homogeneous group, and the school context facilitates access to adult professionals who are available to collaborate across different waves of data collection. Despite slight differences in the sociodemographic characteristics of this subsample versus the general sample of the study, there were no statistically significant differences across the outcome variables assessed (i.e., SECAB-A scales; see Table 1).

**Table 1.** Participants’ Sociodemographic Characteristics and Perceived SEC (Percentage of the Most Frequent Category, Mean and Standard Deviation) and Group Comparisons.

Variable	Total sample (*N* = 796)			Subsample (*n* = 63)			Group differences		
	%	*M*	*SD*	%	*M*	*SD*	*Statistic*	*p*	95% CI
Gender (Female)	80.70			92.10			5.52^a^	.019	
Age		35.72	12.84		46.97	5.31	17.58^b^	< .001	[−14.63, −11.68]
Highest educational qualification (Master’s degree)	36.70			69.80			-8.66^c^	< .001	
Area of residence (Lisbon Metropolitan Area)	73.70			100.00			0.29^d^	< .001	
Perceived SEC									
Self-regulation		3.61	0.57		3.49	0.56	1.40^b^	.170	[−.05, .26]
Self-awareness		4.00	0.47		3.98	0.45	1.11^b^	.272	[−.05, .17]
Conflict management		3.75	0.49		3.70	0.45	0.96^b^	.342	[−.08, .23]
Positive relationship		3.90	0.47		3.78	0.43	1.20^b^	.239	[−.06, .22]
Responsible decision making		3.82	0.49		3.77	0.48	1.11^b^	.277	[−.07, .24]

*Note.* SEC = Social and Emotional Competence; *SD* = standard deviation.

a Chi-square test. ^b^ Robust independent samples t-test. ^c^ Mann–Whitney *U* test. ^d^ Cramer’s V.

### Measures

#### Social and Emotional Competence Assessment Battery for Adults

The SECAB-A is a Portuguese self-report instrument that aims to assess adults’ perception of their social and emotional competence. A deductive content analysis approach following Bardin’s (1977) guidelines supported the items’ generation process. Based on the SEL theoretical framework and research review (Durlak et al., 2015; Elias et al., 1997), five categories corresponding to the previously identified core SEC (i.e., self-awareness, self-regulation, positive relationship, conflict management, and responsible decision making) and respective subcategories regarding specific skills within each core SEC were established a priori. A review of the existing scales and intervention guides covering the different dimensions of SEL integrated the *corpus* to be analyzed. Following the American Psychological Association (APA) Guidelines for Psychological Assessment and Evaluation (American Psychological Association, 2020), the existing scales and intervention guides were selected based on (a) their theoretical framework (if it was to some degree in line with the SEL framework) and (b) a review of prior research on the scales’ validity and reliability, targeted sample (i.e., adults), and cultural adequacy to better match the Portuguese contextual and cultural characteristics. For the encoding and categorization process, the items integrating the *corpus* were deemed the recording unit to be considered. The categorization process comprised two points in time. First, the guides developed in accordance with the SEL framework (i.e., *SSEIC*, Yoder, 2014; *TOOL*, CASEL, 2019) were categorized. These were selected as they were the only ones following the SEL framework and covering all five core SEC domains, thus providing relevant record units for all the categories. The remaining *corpus* was then categorized. For this stage, the existing measures identified in the literature review process were considered as, despite their limitations, they manage to address some SEC domains, and research on their psychometric properties has already been published (i.e., EQ-I, Bar-On, 1997; *ESCQ*, Takšić et al., 2009; *ICQ*, Buhrmester et al., 1988; *QDE-A*, Pérez-Escoda et al., 2010; *TMMS*, Salovey et al., 1995). In this analysis, record units (i.e., items) were categorized whenever they added relevant information, were not redundant to the conceptualization of the subcategory, and were strong items (e.g., the item “Saying ‘no’ when a date/acquaintance asks you to do something you don’t want to do.” from the ICQ, Buhrmester et al., 1988 was considered as *Assertiveness* is an important aspect of *Open communication*). Record units were excluded if they did not express an SEC in accordance with the SEL theoretical framework (e.g., *happiness* from the EQ-i, Bar-On, 1997), were related to specific contexts (*initiate intimate relationships* from the *ICQ*, Buhrmester et al., 1988), or did not add relevant information/were redundant to the already identified subcategories (i.e., revealed theoretical saturation of data). As the record units underlying the analysis corpus were originally written in English and Spanish, a translation process was conducted to ensure linguistic equivalence and cultural adequacy. We followed the APA Guidelines for Psychological Assessment and Evaluation (American Psychological Association, 2020) and the International Test Commission’s (2017) guidelines. Therefore, the record units were primarily translated from English or Spanish to Portuguese by two independent researchers with a master’s degree in educational psychology, in-depth knowledge of the Portuguese culture, and fluent in the three languages (native speakers of Portuguese). A Portuguese researcher with a PhD in educational psychology and fluent in the three languages confronted the translated and adapted record units with the original ones. This researcher identified and discussed discrepancies with the two independent researchers until a consensus was achieved and a single Portuguese version of the record units was created. These translations focused on functional and not literal equivalence (International Test Commission, 2017). In a later stage, the Portuguese version of the record units was back-translated by two bilingual researchers (respectively into English or Spanish), and the original and back-translated versions of the record units were compared and deemed similar (American Psychological Association, 2020; International Test Commission, 2017). Finally, the inferential process/interpretation of the data led to the final items’ formulation. Each subcategory and the coded record units were analyzed, and a prospectus sentence was defined for each specific skill dimension. At least one item was then generated to assess the specific skill dimensions identified and included in the prospectus. This process led to an initial pool of 42 items originally written in Portuguese and covering the five key-competences and specific skills of the SEL framework proposed by Elias et al. (1997). Reverse-coded items were included to minimize the risk of acquiescent response bias (Rattray & Jones, 2007). The described content analysis approach which underlies the item generation process is presented in Table 2.

**Table 2.** Content Analysis Results That Led to the Initial Pool of SECAB-A Items.

SEC domain	Specific skills within the scope of SEC domains	Units of record and respective source	Formulated item for the SECAB-A
1. Self-regulation (Ability to effectively regulate emotions and behaviors; Durlak et al., 2015)	1.1 Emotional and behavioral regulation	1.1.1 To effectively regulate one’s emotions on a daily basis. - “I find ways to manage my emotions and channel them in useful ways without harming anyone.” (TOOL; CASEL, 2019) - “I worry about being in a good mood.” (TMMS; Salovey et al., 1995) - “To effectively and constructively manage/control emotions.” (EQ-i; Bar-On, 1997) - “Sé como generar ocasiones para experimentar emociones agradables/positivas.” (QDE-A; Pérez-Escoda et al., 2010)	Item02. “Sou capaz de regular as minhas emoções de forma eficaz.” (Free translation: “I am able to effectively regulate my emotions.”)
		1.1.2 To effectively regulate one’s emotions and behavior in a moment of crisis. - “I stay calm, clear-headed, and unflappable under high stress and during a crisis.” (TOOL; CASEL, 2019) - “If I find myself getting mad, I try to calm myself down.” (TMMS; Salovey et al., 1995) - “I effectively use multiple strategies (e.g., breathing techniques and mindfulness) when I have a strong emotional reaction.” (SSEIC; Yoder, 2014) - “Acostumbro a moderar mi reacción cuando tengo una emoción fuerte.” (QDE-A; Pérez-Escoda et al., 2010) - “I am able to maintain a good mood even if something bad happens.” (ESCQ; Takšić et al., 2009)	Item06. “Durante momentos de elevado stress, consigo manter a calma.” (Free translation: “In stressful moments, I can stay calm.”)
	1.2 Goal setting and achieving	1.2.1 To establish SMART goals and persevere to achieve them. - “I am pragmatic, setting measurable, challenging, and attainable goals.” (TOOL; CASEL, 2019) - “I continuously refine my personal goals.” (SSEIC; Yoder, 2014) - “To strive to achieve personal goals and actualize one’s potential.” (EQ-i; Bar-On, 1997) - “Tengo claro para qué quiero seguir vivendo.” (QDE-A; Pérez-Escoda et al., 2010)	Item08. “Estabeleço adequadamente os meus objetivos (ex. concretos, mensuráveis, alcançáveis).” (Free translation: “I properly establish my goals [e.g., concrete, measurable, achievable].”)
			Item10. “Monitorizo o meu progresso na persecução dos meus objetivos.” (Free translation: “I monitor my progress toward achieving my goals.”)
	1.3 Self-efficacy	1.3.1 To believe in one’s ability to effectively behave to achieve goals when facing unexpected challenges. - “I feel confident that I can handle whatever comes along with calm self-assurance and a relaxed presence.” (TOOL; CASEL, 2019) - “If I really want to, I will solve a problem that may seem insoluble.” (ESCQ; Takšić et al., 2009)	Item12. “Acredito que sou capaz de lidar com as adversidades que possam surgir.” (Free translation: “I believe that I am capable of dealing with unexpected challenges/obstacles.”)
	1.4 Adaptability	1.4.1 To welcome change and being able to adapt in the face of new information or situations. - “I modify my thinking in the face of new information and realities.” (TOOL; CASEL, 2019) - “I accept new challenges and adjust to change.” (TOOL; CASEL, 2019) - “To adapt and adjust one’s feelings and thinking to new situations.” (EQ-i; Bar-On, 1997) - “Me asustan los cambios.” (QDE-A; Pérez-Escoda et al., 2010)	Item13. “Consigo adaptar-me (ex. pensar de outra forma) em resposta a novas informações ou situações.” (Free translation: “I can adapt [e.g., thinking differently] in response to new information or situations.”)
	1.5 Optimism	1.5.1 To anticipate/identify positive outcomes/aspects even in negative situations. - “I can see the positive even in negative situations.” (TOOL; CASEL, 2019) - “To be positive and look at the brighter side of life.” (EQ-i; Bar-On, 1997)	Item14. “Procuro identificar aspetos positivos mesmo em situações negativas.” (Free translation: “I try to identify positive aspects even in negative situations.”)
	1.6 Organizational skills	1.6.1 To respond to different demands/tasks maintaining focus/energy (through emotional, behavioral and motivational regulation). - “I stay focused and consistent when I work.” (SSEIC; Yoder, 2014) - “I can juggle multiple demands without losing focus or energy.” (TOOL; CASEL, 2019)	Item15. “Sou capaz de conjugar várias tarefas sem perder o foco.” (Free translation: “I can respond to different tasks without losing focus.”)
		1.6.2 To be able to balance and respond to both work and personal life commitments. - “I balance my work life with personal renewal time.” (TOOL; CASEL, 2019)	“Tenho dificuldade em equilibrar o tempo de trabalho e o meu tempo pessoal.” (Free translation: “I struggle to balance work and personal time.”) Note: This item was removed after expert analysis regarding relevance for the construct. Work-life balance emerges on the literature as a consequence of SEC and, particularly, self-regulation.
2. Self-awareness (Ability to understand one’s own emotions, beliefs, and values, and to have accurate self-perception; Durlak et al., 2015)	2.1 Emotional self-awareness	2.1.1 To be able to accurately recognize, identify and name one’s emotions. - “I am able to identify, recognize, and name my emotions in the moment.” (TOOL; CASEL, 2019) - “I pay much attention to my feelings.” (TMMS; Salovey et al., 1995) - “To be aware of and understand one’s emotions.” (EQ-i; Bar-On, 1997) - “Sé poner nombre a las emociones que experimento.” (QDE-A; Pérez-Escoda et al., 2010) - “Putting my feelings and emotions into words comes easily to me.”/“I am capable to list the emotions that I am currently experiencing.” (ESCQ; Takšić et al., 2009)	Item01. “No meu dia-a-dia, sou capaz de identificar e nomear o que estou a sentir no momento.” (Free translation: “In my daily life, I am able to identify and name my emotions when they occur.”)
		2.1.2 To be able to accurately recognize one’s feelings and emotional expressions (physiological, cognitive, and behavioral) toward other people and everyday situations. - “I recognize the relationship between my feelings and my reactions to people and situations.” (TOOL; CASEL, 2019) - “Conozco bien mis emociones.” (QDE-A; Pérez-Escoda et al., 2010) - “Puedo describir fácilmente mis sentimentos.” (QDE-A; Pérez-Escoda et al., 2010) - “I can recognize most of my feelings.” (ESCQ; Takšić et al., 2009) - “I can make sense out of my feelings.” (TMMS; Salovey et al., 1995)	Item03. “No meu dia-a-dia, sei reconhecer as minhas reações emocionais (ex. fisiológicas, cognitivas, comportamentais) no momento.” (Free translation: “In my daily life, I recognize my emotional reactions [e.g., physiological, cognitive, behavioral] at the moment.”)
			Item07. “Estou consciente do que sinto sobre as pessoas ou situações quotidianas.” (Free translation: “I am aware of how I feel about other people or everyday situations.”)
	2.2 Accurate self-perception	2.2.1 To accurately perceive, understand, and accept one’s individual characteristics even if they are not positive (e.g., needs, biases, values, beliefs). - “I know and am realistic about my strengths and limitations.” (TOOL; CASEL, 2019) - “I am aware of what I need to improve upon and grow professionally.” (SSEIC; Yoder, 2014) - “To accurately perceive, understand and accept oneself.” (EQ-i; Bar-On, 1997)	Item11. “Tenho uma perceção realista de quais são as minhas limitações.” (Free translation: “I have an accurate perception of my limitations.”)
			“Tenho dificuldade em identificar quais são os meus pontos fracos.” (Free translation: “I have difficulty identifying my weaknesses.”) Note: This item was removed after expert analysis regarding relevance for the construct. This item was perceived to overlap with Item11 but being harder to comprehend and unclear. Thus, to maintain parsimony this item was deleted from the SECAB-A.
		2.2.2. To be aware of how one’s individual characteristics (e.g., needs, biases, values, beliefs) affect one’s behavior and decisions. - “I know how my own needs, biases, and values affect the decisions I make.” (TOOL; CASEL, 2019) - “I am usually aware of how my emotions, culturally grounded beliefs, and background are precursors to my emotional reactions.” (SSEIC; Yoder, 2014)	Item04. “Sei de que forma o que estou a sentir se reflete nos meus comportamentos (ex. para com as outras pessoas ou situações).” (Free translation: “I know how my feelings impact my behavior [e.g., toward other people or situations].”)
			Item09. “Sei de que forma as minhas características pessoais (ex. necessidades, preconceitos, valores, crenças) afetam as decisões que tomo.” (Free translation: “I know how my personal characteristics [e.g., needs, biases, values, beliefs] affect the decisions I make.”)
		2.2.3 To be aware of how other people’s behavior affect one’s emotions and behavior. - “I understand how student responses (positive and negative) affect my emotions and my behaviors.” (SSEIC; Yoder, 2014)	Item05. “Compreendo como o comportamento das outras pessoas (positivo e negativo) afeta o que sinto e como reajo.” (Free translation: “I understand how other people’s behavior [positive or negative] affect my emotions and behaviors.”)
3. Conflict management (Ability to effectively manage conflict situations and negative social interactions; Durlak et al., 2015)	3.1 Taking perspective and appreciate diversity	3.1.1 To take perspective/be able to comprehend different perspectives, opinions, needs, or behaviors. - “Acepto y respeto que los otros piensen y actúen de forma diferente a mí.” (QDE-A; Pérez-Escoda et al., 2010)	Item01. “Tenho dificuldade em compreender perspetivas diferentes da minha.” (Free translation: “I have difficulty in understanding perspectives other than mine.”)
		3.1.2 To appreciate, respect, and value diverse backgrounds, perspectives, and cultures. - “I try to understand the perspective and experiences of others before I offer suggestions.” (TOOL; CASEL, 2019) - “I address the commonalities and differences (e.g., racial, ethnic, cultural) that exist among students.” (SSEIC; Yoder, 2014) - “I appreciate and get along with people of diverse backgrounds and cultures in my school community and utilize inclusionary practices to ensure all voices are represented.” (TOOL; CASEL, 2019)	Item11. “Tento entender a perspetiva e as experiências dos outros antes de oferecer sugestões.” (Free translation: “I try to understand others’ perspectives and experiences before offering suggestions.”)
	3.2 Empathy	3.2.1 To be empathic even in the face of different approaches/interpretations of reality. - “Being able to show genuine empathetic concern even when a companion’s problem is uninteresting to you.” (ICQ; Buhrmester et al., 1988) - “Sé ponerme en el lugar de los otros para comprenderlos bien.” (QDE-A; Pérez-Escoda et al., 2010)	Item03. “Sou capaz de mostrar empatia genuína mesmo quando não me identifico com o problema da outra pessoa.” (Free translation: “I am able to show genuine empathy even when I don’t identify with the other person’s problem.”)
	3.3. Openly admit one’s mistakes	3.3.1 To be able to openly admit personal mistakes/misbehaviors and to be receptive to others’ criticism/feedback. - “I am able to openly admit my mistakes and shortcomings to myself and others.” (TOOL; CASEL, 2019) - “Me siento herido fácilmente cuando los otros critican mi conducta o trabajo.” (QDE-A; Pérez-Escoda et al., 2010)	Item07. “Tenho dificuldade em admitir abertamente os meus erros perante as outras pessoas.” (Free translation: “I find it difficult to openly admit my mistakes to other people”)
	3.4 Conflict management	3.4.1 When facing conflict, being able to recognize that other people have a valid point, and one’s might be wrong. - “When angry with a companion, being able to accept that s/he has a valid point of view even if you don’t agree with that view.” (ICQ; Buhrmester et al., 1988) - “Being able to admit that you might be wrong when a disagreement with a close companion begins to build into a serious fight.” (ICQ; Buhrmester et al., 1988)	Item08. “Durante uma discussão, sou capaz de admitir que a outra pessoa tem razão.” (Free translation: “During an argument, I can admit that the other person is right.”)
		3.4.2 When facing conflict, to act without resentment. - “Being able to put begrudging (resentful) feelings aside when having a fight with a close companion.” (ICQ; Buhrmester et al., 1988)	Item14. “Sou capaz de deixar de lado ressentimentos para melhor resolver um conflito.” (Free translation: “I am able to let go of resentments to better resolve a conflict.”)
		3.4.3 When facing conflict, to be able to active listening others perspectives/vision of the situation. - “I am comfortable dealing with conflict, listening to feelings from all parties and helping them understand different perspectives.” (TOOL; CASEL, 2019) - “I try to understand why my students are or are not actively participating (. . .)” (SSEIC; Yoder, 2014) - “When having a conflict with a close companion, really listening to his or her complaints and not trying to ‘read’ his/her mind.” (ICQ; Buhrmester et al., 1988)	Item15. “Perante um conflito com alguém conhecido, sou capaz de ouvir atentamente o que essa pessoa me diz em vez de tentar ‘ler’ a sua mente.” (Free translation: “In the face of a conflict with someone I know, I am able to listen carefully to what that person is saying to me rather than trying to ‘read’ their mind.”)
		3.4.4 To effectively manage conflicts by looking for collaboration/search for common solutions. - “Me bloqueo cuando tengo que resolver conflictos.” (QDE-A; Pérez-Escoda et al., 2010) - “I am able to guide conflicting parties to find a common solution.” (TOOL; CASEL, 2019)	“Sou capaz de gerir conflitos adequadamente, ouvindo todas as partes e procurando uma solução de compromisso perante diferentes perspetivas.” (Free translation: “I am able to manage conflicts properly, listening to all parties and looking for a compromise solution in the face of different perspectives.”) Note: This item was removed after expert analysis regarding relevance for the construct. This item was perceived to overlap with the Problem-Solving (item03) skills within Responsible Decision Making which consider the search for compromise solutions. Moreover, the content source underlying this item is more general and better explained by the remaining items of the scale.
	3.5 Respect for others	3.5.1 To be able to respect others, communicate with focus on others’ behavior and expect their best. - “Being able to work through a specific problem with a companion without resorting to global accusations (‘you always do that’).” (ICQ; Buhrmester et al., 1988) - “I believe that, in general, people are doing their best, and I expect the best of them.” (TOOL; CASEL, 2019)	Item16. “Sou capaz de resolver um problema específico com alguém sem recorrer a acusações globais (ex. ‘fazes sempre isso,’ ‘nunca fazes isso’).” (Free translation: “I am able to solve a problem with someone without resorting to global accusations [e.g., ‘you always do this’, ‘you never do this’].”)
			“Acredito que, em geral, as pessoas dão o melhor de si/fazem o seu melhor.” (Free translation: “I believe that, in general, people give their best/do their best.”) Note: This item was removed after expert analysis regarding relevance for the construct. This item was perceived to do not directly express and add to the conceptualization of Respect for others. This item was evaluate as referring to a general belief about others and not as expressing a competence reflecting Respect for others. Also, the alternative Item16 was perceived to be clearer and more appropriate.
4. Positive relationship (Ability to build and maintain strong and supportive relationships and interact effectively with others; Durlak et al., 2015)	4.1 Open communication	4.1.1 To be able to foster emotionally safe and engaging environments that promote mutually satisfying relationships. - “I foster an emotionally nurturing and safe environment for staff, students, families, and community members.” (TOOL; CASEL, 2019) - “I successfully support positive emotions and respond to negative emotions.” (SSEIC; Yoder, 2014) - “To establish mutually satisfying relationships and relate well with others.” (EQ-i; Bar-On, 1997)	Item02. “Sou capaz de criar um ambiente emocionalmente seguro e envolvente para as pessoas com quem me relaciono.” (Free translation: “I am able to create an emotionally safe and engaging environment for the people I interact with.”)
		4.1.2 To express one’s values, goals, perspectives and expectations authentically. - “I am open and authentic with others about my values and beliefs, goals, and guiding principles.” (TOOL; CASEL, 2019) - “To effectively and constructively express one’s emotions and oneself.” (EQ-i; Bar-On, 1997) - “I clearly communicate behavioral and academic expectations (. . .)” (SSEIC; Yoder, 2014)	Item04. “Sou aberto/a e autêntico/a com os outros sobre as minhas características pessoais (ex. valores, crenças, objetivos, princípios orientadores).” (Free translation: “I am open and authentic with others about my personal characteristics [e.g., values, beliefs, goals, guiding principles].”)
		4.1.3 To be able to say no and communicate with assertiveness. - “Saying ‘no’ when a date/acquaintance asks you to do something you don’t want to do.” (ICQ; Buhrmester et al., 1988) - “When something doesn’t suit me, I show this immediately.” (ESCQ; Takšić et al., 2009)	Item09. “Sou capaz de dizer ‘não’ quando me pedem para fazer algo com que não concordo/vai contra o que acredito.” (Free translation: I can say ‘no’ when asked to do something I don’t agree with/goes against what I believe.)
		4.1.4 To openly communicate to others what their actions make one’s feel. - “Telling a companion that he or she has done something to hurt your feelings.” (ICQ; Buhrmester et al., 1988) - “Me cuesta defender opiniones diferentes a la de las otras personas.” (QDE-A; Pérez-Escoda et al., 2010) - “When I don’t like a person, I find ways to let him/her know.” (ESCQ; Takšić et al., 2009)	Item10. “Sou capaz de dizer abertamente a outras pessoas o que as suas ações me fazem sentir (positiva ou negativamente).” (Free translation: “I can openly tell other people what their actions make me feel [good or bad].”)
	4.2 Building relationships	4.2.1 To be able to give constructive feedback to others. - “I give timely and constructive feedback as a coach and mentor.” (TOOL; CASEL, 2019)	Item05. “Dou feedback adequado (ex. oportuno, construtivo).” (Free translation: “I give appropriate feedback [e.g., timely, constructive].”)
		4.2.2 To pay attention, be aware and recognize others’ emotions through the observation of their (non-)verbal communication. - “I listen actively and can grasp another person’s perspective and feelings from both verbal and nonverbal cues.” (TOOL; CASEL, 2019) - “I usually understand the perspectives of my students and can pay attention to their emotional cues during classroom interactions.” (SSEIC; Yoder, 2014) - “To be aware of and understand how others feel.” (EQ-i; Bar-On, 1997) - “Me resulta fácil darme cuenta de cómo se sienten los otros.”/“Me resulta difícil saber como se sienten los otros.” (QDE-A; Pérez-Escoda et al., 2010) - “When I meet an acquaintance, I immediately notice his/her mood.”/“If I observe a person in the presence of others, I can determine precisely her or his/her emotions.”/“I am able to tell somebody’s feelings by the expression on his/her face.” (ESCQ; Takšić et al., 2009)	Item06. “Consigo compreender o que os outros estão a sentir através da forma como comunicam (ex. comunicação verbal, comunicação não verbal).” (Free translation: “I am able to understand others’ feelings through the way they communicate [e.g., verbal communication, non-verbal communication].”)
	4.3 Organizational awareness	4.3.1 To comprehend and act in accordance with social norms. - “I understand the organizational forces at work, guiding values, and unspoken rules that operate among people.” (TOOL; CASEL, 2019)	Item12. “Compreendo os valores e as regras implícitas que estão presentes na interação com as outras pessoas.” (Free translation: “I understand the values and implicit rules that are present in social interactions.”)
	4.4 Teamwork and collaboration	4.4.1 To effectively work in a team and promote collaboration between all parties. - “I am good at teamwork and collaboration and generate a collegial atmosphere that inspires us all.” (TOOL; CASEL, 2019) - “I involve key stakeholders in important decision-making tasks to ensure we are making wise choices.” (TOOL; CASEL, 2019)	Item13. “Procuro trabalhar em equipa, colaborativamente, para a resolução de problemas.” (Free translation: “I try to work in a team, collaboratively, to solve problems.”)
5. Responsible decision-making (Ability to make ethical and constructive decisions, evaluate and reflect on personal behavior, and effectively solve problems; Durlak et al., 2015)	5.1 Problem identification and situation analysis	5.1.1 In the face of a problem, to be able to generate multiple solutions and anticipate their outcomes. - “I am able to define the core of the problem and differentiate it from solution options.” (TOOL; CASEL, 2019) - “I involve others to generate multiple solutions and predict the outcome (of each solution) for key problems.” (TOOL; CASEL, 2019) - “Me resulta fácil pensar en las consecuencias de mis decisiones.” (QDE-A; Pérez-Escoda et al., 2010)	Item01. “Quando tenho um problema, consigo pensar em várias soluções alternativas.” (Free translation: “When I have a problem, I can think of alternative solutions.”)
			Item02. “Perante soluções alternativas para um problema, sou capaz de antecipar o resultado de cada uma delas antes de decidir.” (Free translation: “Faced with alternative solutions to a problem, I am able to anticipate the outcomes before deciding.”)
			“Consigo identificar com clareza os meus problemas.” (Free translation: “I am able to identify my problems with clarity.”) Note: This item was removed after expert analysis regarding relevance for the construct. This item was perceived to be less relevant in light with the subcategory prospectus. To maintain parsimony the item was removed, since the ability to generate multiple solutions (Item01) and to anticipate their outcomes (Item02) were perceived more relevant and sufficient.
	5.2 Problem-solving	5.2.1 To be able to effectively solve problems, including all parties involved to find a common solution. - “To effectively solve problems of a personal and interpersonal nature.” (EQ-i; Bar-On, 1997) - “I am effective at considering multiple forms of evidence, such as balancing the needs and the behaviors of my entire class.” (SSEIC; Yoder, 2014) - “I regularly include my students and/or collaborate with colleagues to solve problems that arise in the classroom.” (SSEIC; Yoder, 2014)	Item03. “Na presença de um problema que afeta também outras pessoas, procuro envolver todas as partes na procura da solução.” (Free translation: “In the presence of a problem that also affects other people, I try to involve all parties to find a common solution.”)
		5.2.2 To be able to ethically and respectfully, make and communicate decisions even when unpopular. - “I find practical and respectful ways to overcome barriers, even when it comes to making decisions that may not be popular.” (TOOL; CASEL, 2019)	Item04. “Mesmo quando se trata de tomar decisões que podem não ser populares, encontro formas respeitosas de superar os problemas.” (Free translation: “Even when it comes to making decisions that may not be popular, I find respectful ways to overcome barriers.”)
	5.3 Behavior evaluation and reflection	5.3.1 To create opportunities for reflection on progress toward goals. - “I provide opportunities for self-reflection and group reflection on progress toward goals and the process used.” (TOOL; CASEL, 2019)	Item05. “Crio oportunidades de reflexão (ex. autorreflexão, reflexão em grupo) para monitorizar o progresso em direção aos objetivos estabelecidos.” (Free translation: “I create opportunities for reflection [e.g., self-reflection, group reflection] to monitor progress toward established goals.”)
	5.4 Personal, moral, & ethical responsibility	5.4.1 To threat other people respectfully with personal, social and moral responsibility. - “I treat other people in the way I would want to be treated.” (TOOL; CASEL, 2019)	Item06. “Trato as outras pessoas da maneira que gostaria de ser tratado/a.” (Free translation: “I treat other people the way I would like to be treated.”)

*Note.* SEC = Social and Emotional Competence; SECAB-A = Social and Emotional Competence Assessment Battery for Adults; CASEL = Collaborative for Academic, Social, and Emotional Learning consortium; TMMS = Trait Meta-Mood Scale; QDE-A = Questionari de Desarrollo Emocional para Adultos; SSEIC = Self-Assessing Social and Emotional Instruction and Competencies; ESCQ = Emotional Skills and Competence Questionnaire; ICQ = Interpersonal Competence Questionnaire.

To assure facial/content validity, adding to the initial item generation process, which was supported by the review of associated literature, both a panel of independent experts in the field and a group of proposed respondents from the target population were consulted for a try out of the measure (International Test Commission, 2017; Rattray & Jones, 2007). Primarily, the initial pool of 42 items was evaluated by two independent judges who were educational psychologists with expertise in the SEL field of research and native Portuguese speakers. Experts were asked to read the items and rate each one of them from 1 (*Does not meet the criteria*) to 4 (*Meets the criteria at a high level*) regarding their *Clarity* (i.e., whether the item was easily understood and was suited to the Portuguese context), *Construction* (i.e., whether the item had adequate syntax and semantics), and *Relevance* to the proposed dimension (i.e., whether the item had a logical relationship with the dimension it was measuring and was essential/important and should therefore be included); scales were rated for *Sufficiency/Parsimony* of the dimensions (i.e., whether the items belonging to the same scale were sufficient to obtain the measurement of this dimension and the scale was parsimonious; adapted from Escobar-Pérez & Cuervo-Martínez, 2008). Whenever an item/scale was rated below 4, the experts were asked to comment on their understanding of what alterations were required. Overall, the experts perceived the items as clear and easy to understand, coherent, and relevant; nevertheless, their suggestions (e.g., wording and rephrasing) were integrated to improve the items. In this process, after discussing the comments/evaluations of the experts, five items that were evaluated with scores below 3 in the relevance dimension by the judges were dropped from the initial pool (See Table 2 for detailed information on the items removed). The five scales were perceived as sufficient and parsimonious. The revised pool of 37 items and the instructions were analyzed for clarity by five Portuguese adults who rated the items as clear and easy to understand.

The final version of the SECAB-A, presumably addressing the specific skills across the five key domains of SEC and following the SEL framework proposed by Elias et al. (1997), was then composed of three independent questionnaires with a total of 37 revised items. The first questionnaire assessing *intrapersonal skills* consisted of two scales, one regarding *self-awareness* (i.e., seven items related to emotional self-awareness and accurate self-perception; e.g., “In my daily life, I am able to identify and name my emotions when they occur.”) and the other concerning *self-regulation* (i.e., eight items addressing emotional and behavioral regulation, goal setting and achieving, adaptability, self-efficacy, optimism and organizational skills; e.g., “I can adapt [e.g., thinking differently] toward new information or situations.”). The second questionnaire measuring *interpersonal skills* also included two scales, the first related to *positive relationship* (i.e., eight items related to the establishment and maintenance of strong and supportive interpersonal relationships through open and clear communication, give constructive feedback, organizational awareness, team work and collaboration; e.g., “I can say ‘no’ when asked to do something I don’t agree with/goes against what I believe.”) and the second pertaining to *conflict management* (i.e., eight items related to taking perspective, appreciating diversity, be empathic, respect others, communicate mistakes openly, active listening and conflict management; e.g., “I try to understand others’ perspective and experiences before offering suggestions.”). Finally, the SECAB-A integrates a unidimensional questionnaire measuring *responsible decision-making* (i.e., six items concerning problem identification and solving, behavior evaluation and reflection, and personal, moral, and ethical responsibility; e.g., “In the presence of a problem that also affects other people, I try to involve all parties in finding the solution.”).

The participants were asked to evaluate how each item, across the questionnaires, characterized their own behavior on a 5-point *Likert-type* scale (from 1—*Never or hardly ever* to 5—*Almost always or always*). Frequency items were chosen as they are considered more informative and reliable when specific behaviors are being assessed (Marfeo et al., 2014; Rattray & Jones, 2007). The length of the rating scale was defined according to previous research indication (Smith et al., 2003) and despite the lack of a consensus, a mid-point was maintained since its absence may increase nonresponse bias (Rattray & Jones, 2007).

#### Positive and Negative Affect Schedule

Positive affect was evaluated through the *Positive affect* scale of the Positive and Negative Affect Schedule (PANAS) questionnaire (Watson et al., 1988; Portuguese version by Galinha & Pais-Ribeiro, 2005). This scale comprises 10 items for which participants were asked to rate how often they had felt each positive emotion over the past 2-week period (e.g., excited, happy, interested; ω_T1_ = 0.89 and ω_T2_ = 0.83). Items were evaluated on a 5-point *Likert-type* scale (from 1—*Very slightly or not at all* to 5—*Extremely*).

#### Emotion Regulation Questionnaire (ERQ)

The *Cognitive reappraisal* scale of the Emotion Regulation Questionnaire (ERQ; Gross & John, 2003; Portuguese version by Vaz & Martins, 2008) was used to evaluate how the participants regulated their emotions when facing different situations (6 items, e.g., “I control my emotions by changing the way I think about the situation I’m in.”; ω_T1_ = 0.81 and ω_T2_ = 0.82). Items were evaluated on a 7-point *Likert-type* scale (from 1—*Strongly disagree* to 7—*Strongly agree*).

#### Maslach’ Burnout Inventory–Educators Survey

Perceived experience of the two core burnout symptoms, *Emotional exhaustion* (nine items, e.g., “I feel emotionally drained by my work.”; ω_T1_ = 0.91 and ω_T2_ = 0.90) and *Depersonalization* (five items, e.g., “I feel students blame me for some of their problems.”; ω_T1_ = 0.76 and ω_T2_ = 0.74), was assessed through the Maslach’ Burnout Inventory–Educators Survey (MBI-ES; Maslach et al., 1996; Portuguese version by Marques-Pinto et al., 2005). The 14 items of the scales used were rated on a 7-point *Likert-type* scale (from 0—*Never* to 6—*Every day*).

#### Sociodemographic Information

Sociodemographic information regarding participants’ gender, age, highest educational level, and geographic area of residence was collected by means of a general questionnaire.

### Procedures

The data were collected online through the *Qualtrics* platform where the pool of 37 items and the sociodemographic questionnaire were uploaded as an online survey. Besides the SECAB-A and the sociodemographic questionnaire, the convenience subsample of 63 participants also responded to the PANAS, ERQ, and MBI-ES measures to allow for the convergent and discriminant validity analysis. Within this 63 participant subsample, data were collected at two points in time, and participants were asked to respond to all the measures a second time after an interval of a month to allow for the test–retest analysis of the SECAB-A. The online survey link was disseminated via email and social networks resulting in a non-probabilistic sampling. Nevertheless, we were able to reach participants from across the country. When accessing the survey link, participants received an explanation of the study’s aims and the informed consent (i.e., guaranteeing voluntary participation and the possibility of dropping out at any moment, and ensuring confidentiality and anonymity of the data). After giving their consent, the participants responded to the questionnaires, with a mean duration of 10 min (and around 20 min for the 63 participants in the subsample). As the data were collected online, in the case of missing values, the software alerted the participants to complete their responses before submission, and hence there were no missing data. Also, participation was only registered when the full data protocol was completed. To directly reduce Social Desirability Bias (SDB), anonymity and confidentiality of responses were ensured, and a statement to encourage honesty was included in the introduction to the survey (Larson, 2019). To improve data validity, consistency of response (e.g., age and educational level combinations), unique IP addresses to exclude multiple submissions, and completion time (a threshold of 5 min was considered) and progress (only responses with 100% completion progress were considered and registered by the software) were checked (Aust et al., 2013). In addition, some sociodemographic data (i.e., age and area of residence) were collected through text entry boxes to facilitate the detection of random answers, spam, or the use of autofill software (Dewitt et al., 2018). Responses that did not meet the data validation protocol criteria were considered invalid and excluded from the sample. The data collection process lasted 6 weeks, and no compensation was offered to the participants.

### Data Analysis

The data analyses were performed using the following software packages designed for R environment (R Core Team, 2018): blandr (Datta, 2017), lavaan (Rosseel, 2012), semTools (2015), semPlot (Epskamp, 2015), psych (Revelle, 2021), psy (Falissard, 2012), GPArotation (Bernaards & Jennrich, 2015), ufs (Peters, 2021), and WRS2 (Mair & Wilcox, 2020). For sample size definition, the indications of Kline (2011) were followed according to which a sample to parameters ratio of 20:1 was ensured. In addition, data diagnostics were performed. More specifically, an adequate correlation between variables without evidence of collinearity was checked using the Bartlett test, Kaiser–Meyer–Olkin (KMO), and variance inflation factor (VIF). Relevant correlation between variables occurred whenever the Bartlett test had an associated *p* value of <.05 and the KMO values were >0.5 (Kaiser, 1974), with variables being redundant when VIF >5 (Menard, 1995). As for outliers and distributional assumptions, graphical representations (quantile–quantile plots) were used with extreme values and deviations from normal distributions being considered when |*z*| > 3 (Kline, 2011).

To test the study hypotheses regarding the number of factors retained for each scale the standardized root mean square residual (SRMR) was used, and the factors retained when the models fell below the recommended cut-off of SRMR <0.08 (Hu & Bentler, 1998). Confirmatory factor analysis (CFA) models were then performed, considering the number of factors previously tested. In addition to the models’ chi-square and SRMR values, their fit was evaluated using additional fit measures such as the Tucker–Lewis fit index (TLI; Tucker & Lewis, 1973), comparative fit index (CFI; Bentler, 1990), and the root mean square error of approximation (RMSEA; Browne & Cudeck, 1992). According to Hu and Bentler (1998), CFI and TLI values >0.90 reveal a good model fit, as well as RMSEA values below 0.08. Also, Akaike information criterion (AIC; Akaike, 1998) and the Bayesian information criteria (BIC; Schwarz, 1978) were used to account for model complexity with models with smaller AIC and BIC values being the most suitable. Model specification analyses were performed, and modification indices (MI; cutoff of >25) were included in the models only when theoretically sustained. CFA solutions were tested against alternative models to evaluate model fit changes and to perform the chi-square difference statistic test.

An additional scale diagnosis was performed to evaluate reliability, as well as convergent and discriminant validities. For score reliability, coefficient omega (McDonald, 1999) was applied. According to Nunnally and Bernstein (1994), internal consistency is considered adequate for values between 0.60 and 0.70 and good when reliability is ≥0.70. Test–retest was also performed with the readministration of the measures under analysis on a second occasion. Pearson correlations, two-way mixed effects intraclass correlation coefficient (ICC; absolute agreement), and Bland–Altman plots were computed to evaluate the association between the sets of scores and assess the test–retest reliability and agreement (Berchtold, 2016). Finally, convergent and discriminant validities were tested against external measures. Evidence of convergent validity occurred when intercorrelations were at least moderate in size, while for discriminant validity correlations should be small (Kline, 2011). According to Cohen (1988), both Pearson correlation coefficients and convergent and discriminant validity indicators should be interpreted as follows: correlation values around 0.10 reflect a small association, while values around 0.30 and 0.50 illustrate moderate and large associations, respectively. For ICC interpretation, values are considered poor if lower than 0.50, moderate when between 0.50 and 0.75, good if between 0.75 and 0.90, and excellent when greater than 0.90 (Koo & Li, 2016). Finally, associations between the sociodemographic variables and the SECAB-A questionnaires were computed through Pearson correlations.

## Results

### Data Diagnostics

Correlations between variables were adequate for each questionnaire (intrapersonal competence questionnaire: χ^2^ (105) = 3,108.03, *p* < .001; KMO_Overall_ = 0.88; KMO_Items_ > 0.80; interpersonal competence questionnaire: χ^2^ (120) = 2,172.00, *p* < .001; KMO_Overall_ = 0.85; KMO_Items_ > 0.70; responsible decision-making competence questionnaire: χ^2^ (15) = 681.85, *p* < .001; KMO_Overall_ = .78; KMO_Items_ > .75; and without evidence of multicollinearity (VIF < 2). The analysis of detrended Q-Q plots suggested minor deviations, with most data points being clustered around 0 and not surpassing 1 standard deviation. Thus, maximum likelihood estimation was used for CFA models.

### Hypothesis Testing: Extraction of Factors and CFA

**Figure 1.**
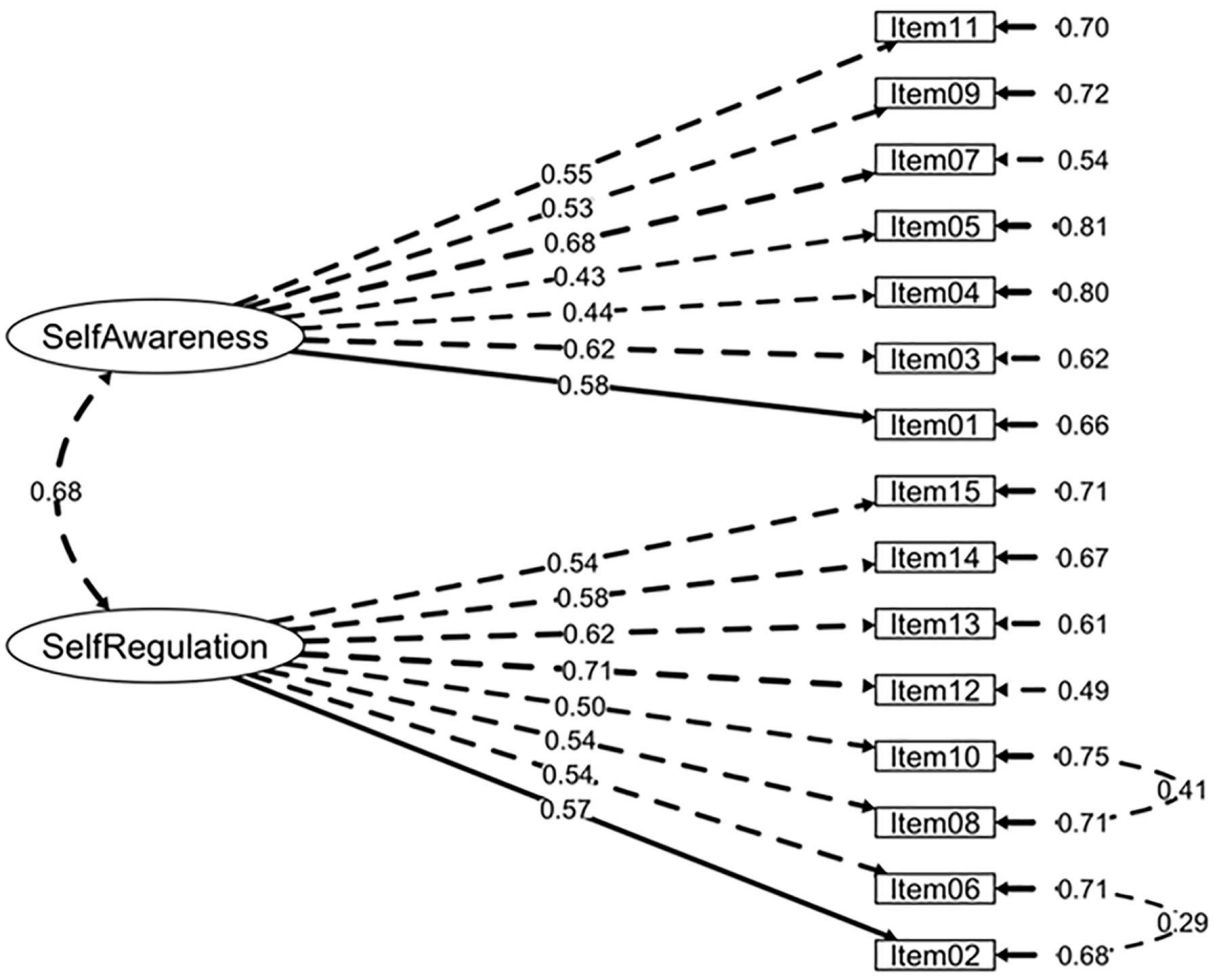
Factor Structure and Factor Loadings of the SECAB-A Intrapersonal Competence Questionnaire *Note.* SECAB-A = Social and Emotional Competence Assessment Battery for Adults.

**Figure 2.**
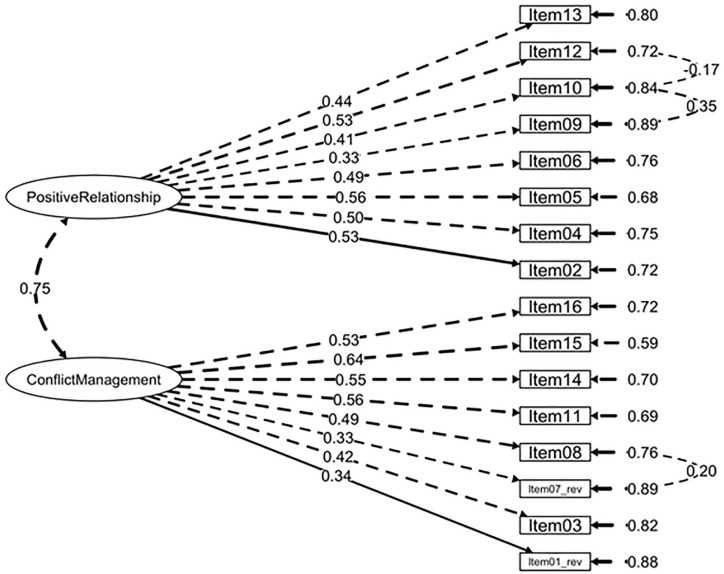
Factor Structure and Factor Loadings of the SECAB-A Interpersonal Competence Questionnaire *Note.* SECAB-A = Social and Emotional Competence Assessment Battery for Adults.

**Figure 3.**
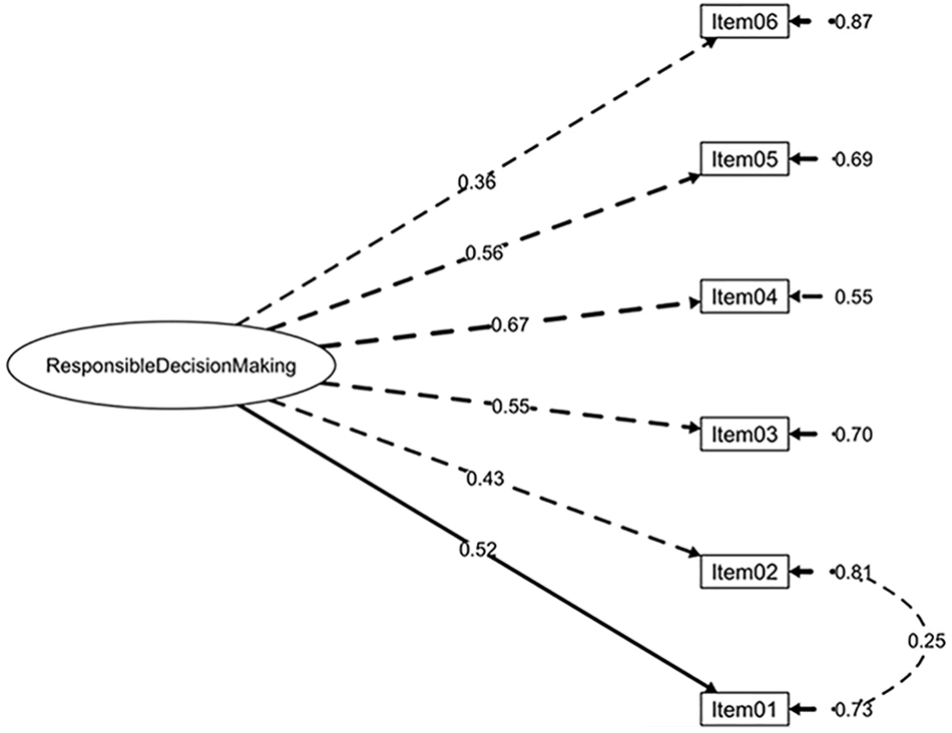
Factor Structure and Factor Loadings of the SECAB-A Responsible Decision-Making Competence Questionnaire *Note.* SECAB-A = Social and Emotional Competence Assessment Battery for Adults.

Extraction results regarding the hypothesized factor solutions were corroborated by initial SRMR values (<0.05). Examination of MI led to the following adjustments to improve the models’ fit: For the *Intrapersonal competence questionnaire*, Items 02 and 06 (i.e., both referring to the emotional regulation dimension) and Items 08 and 10 (i.e., both items assessing the ability to set and achieve goals dimension) were forced to covary. For the *Interpersonal competence questionnaire*, Item 10 was forced to covary with Item 09 and Item 12 (i.e., the three items related to clear communication), and Item 07 reversed and Item 08 (i.e., both items referring to the acknowledgment of personal mistakes dimension) were also forced to covary. Finally, for the *Responsible Decision-Making competence questionnaire*, Items 01 and 02 (i.e., both items addressing a problem-solving dimension) were forced to covary. Table 3 depicts the results for the CFA models under study and alternative solutions, revealing the models’ fit was better for the hypothesized structures. The final factor structures of the three SECAB-A questionnaires are presented in Figures 1, 2, and 3. In addition, an alternative 5 first-order factor model of the SECAB-A was tested, but CFA results revealed a poor fit of the model (χ^2^ = 1,632.66, *df* = 609, *p* < .001, *χ*^2^*/df* = 2.68, CFI = 0.87, TLI = 0.86; SRMR = 0.05, RMSEA = 0.05, 90% CI [0.04, 0.05]).

**Table 3.** Goodness-of-Fit Statistics for the Solutions of the Intrapersonal Competence Questionnaire, the Interpersonal Competence Questionnaire, and the Responsible Decision-Making Competence Questionnaire (N = 796).

Models	χ^2^	*df*	χ^2^/*df*	CFI	TLI	SRMR	RMSEA	90% CI	AIC	BIC	*df*, ∆ χ^2^	Model comparison
CFA for the specified models of the Intrapersonal competence questionnaire (15 items and modification indices)												
Model A	570.812 ***	88	6.49	.84	.81	.06	.08	[.08, .09]	26,479.43	26,527.56	—	—
Model B	337.781 ***	87	3.84	.92	.90	.05	.06	[.05, .07]	26,248.40	26,298.04	*1*, 233.03 ***	Model A
CFA for the specified models of the Interpersonal competence questionnaire (16 items and modification indices)												
Model A	375.580 ***	101	3.72	.87	.84	.05	.06	[.05, .07]	29,728.28	29,780.92	—	—
Model B	275.483 ***	100	2.75	.92	.90	.04	.05	[.04, .05]	29,630.18	29,684.33	*1*, 100.10 ***	Model A
CFA for the specified models of the Responsible Decision-Making competence questionnaire (6 items and modification indices)												
Model A	7.461 ***	8	0.93	1.00	1.00	.02	.00	[.00, .04]	10,457.52	10,477.07	—	—

*Note.* χ^2^ = chi-square test; *df* = degrees of freedom; CFI = Comparative Fit Index; TLI = Tucker–Lewis Index; SRMR = Standardized Root Mean Square Residual; RMSEA = Root Mean Square Error of Approximation; AIC = Akaike Information Criteria; BIC = Bayesian information criteria. Model A (unidimensional structure), Model B (two first-order factors structure); CFA = Confirmatory factor analysis.

*** *p* < .001.

### Reliability, Convergent, and Discriminant Validity

**Figure 4.**
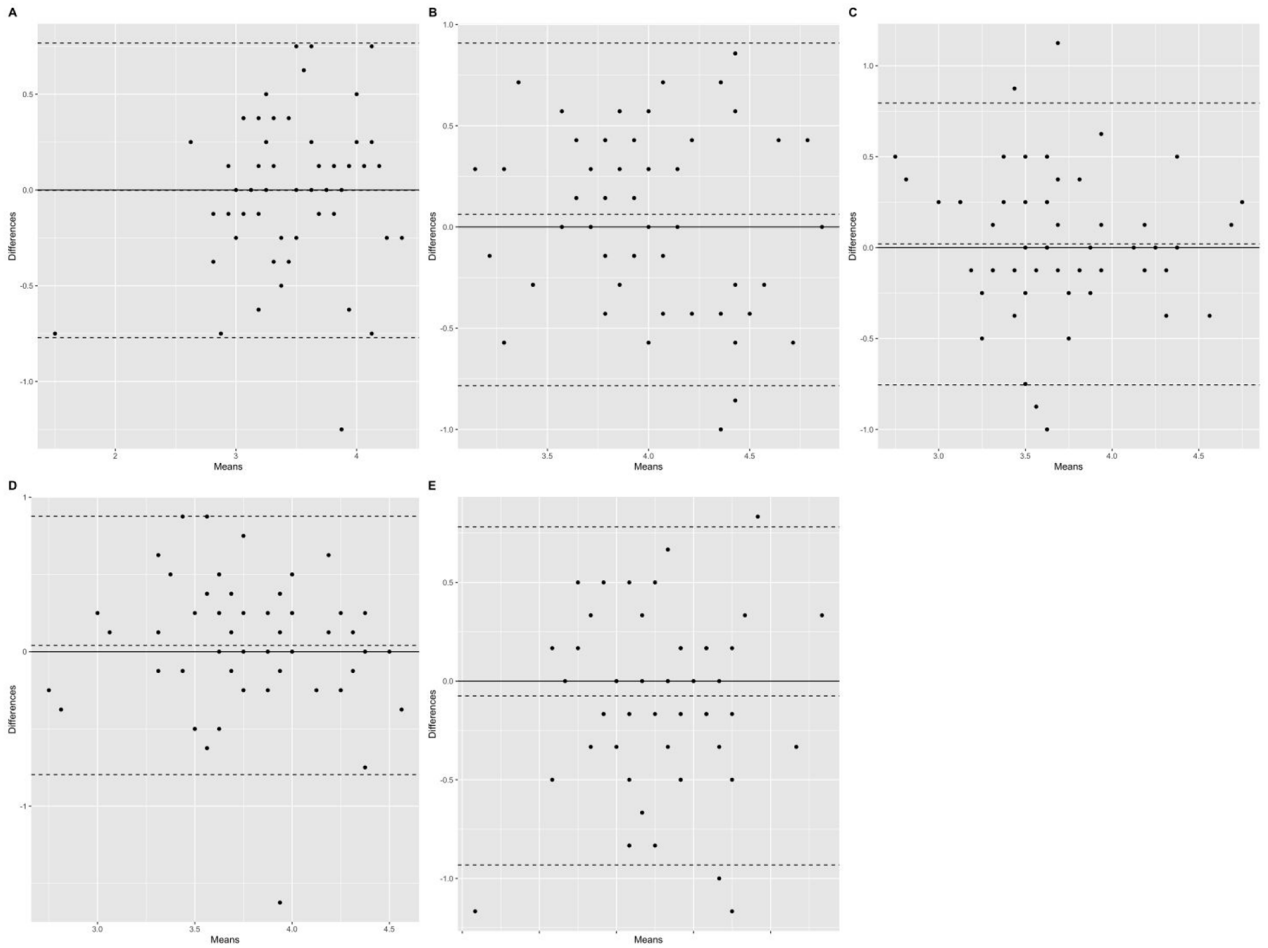
Bland-Altman Plot for Comparison of 2 Methods for the SECAB-A Scales. (A) Self-regulation scale. (B) Self-Awareness Scale. (C) Conflict Management Scale. (D) Positive Relationship Scale. (E) Responsible Decision-Making Scale. *Note*. SECAB-A = Social and Emotional Competence Assessment Battery for Adults.

Table 4 illustrates both the descriptive statistics and internal consistency for the scales under study. Coefficient omegas were adequate and correlations between scales were moderate to large in size. As for test–retest, scores revealed large correlations between the two data collection points, moderate to good ICC coefficients, and the Bland–Altman plots (Figure 4) also revealed adequate variability across time. Associations were large, positive, and statistically significant. Coefficient omegas remained adequate across occasions. As anticipated, the intercorrelations of self-awareness and self-regulation with positive affect and cognitive reappraisal were positive and moderate, suggesting convergent validity (Table 5). As for discriminant validity, intercorrelations between the study measures and burnout measures were small and nonsignificant (Table 6).

**Table 4.** Descriptive (Mean and Standard Deviation), Reliability (ω) and Association (Pearson r) Measures of the Five SECAB-A Scales (N = 796); and Test-Retest Reliability With Reliability (ω_T1_ and ω_T2_), Pearson Correlation (r_T1-T2_) and ICC Coefficient at the Two Points in Time for the Five SECAB-A Scales (n = 63).

Scales	*M (SD)*	Ω [95% CI]	1.	2.	3.	4.	5.	ω _T1_	ω _T2_	*r_T1-T2_*	ICC [95% CI]
1. Self-regulation	3.61 (0.57)	.80 [.77, .83]	—					.85	.82	.73 ***	.84 [.74, .91]
2. Self-awareness	4.00 (0.47)	.75 [.72, .78]	.53 ***	—				.79	.79	.56 ***	.72 [.53, .83]
3. Conflict management	3.75 (0.49)	.71 [.68, .75]	.48 ***	.42 ***	—			.81	.79	.65 ***	.78 [.64, .87]
4. Positive relationship	3.90 (0.47)	.70 [.66, .73]	.58 ***	.60 ***	.50 ***	—		.70	.74	.54 ***	.70 [.51, .82]
5. Responsible decision making	3.82 (0.49)	.70 [.67, .74]	.66 ***	.54 ***	.55 ***	.65 ***	—	.74	.68	.54 ***	.70 [.49, .81]

*Note*. CI = confidence interval; SD = standard deviation; SECAB-A = Social and Emotional Competence Assessment Battery for Adults; ICC = Intraclass Correlation coefficient.

*** *p <* .001.

**Table 5.** Intercorrelation Between SECAB-A Intrapersonal Scales and Positive Affect and Cognitive Reappraisal Measures.

Scales	Self-regulation	Self-awareness
Positive affect	.44 ***	.41 ***
Cognitive reappraisal	.36 ***	.41 ***

*Note.* Positive affect and Cognitive reappraisal are, respectively, scales from the PANAS and ERQ measures used. SECAB-A = Social and Emotional Competence Assessment Battery for Adults.

*** *p* < .001.

**Table 6.** Intercorrelation Between SECAB-A Scales and Emotional Exhaustion and Depersonalization Scales.

Scales	Self-regulation	Self-awareness	Conflict management	Positive relationship	Responsible decision-making
Emotional exhaustion	−.39***	−.16	−.12	−.21	−.20
Depersonalization	−.19	−.15	−.35***	−.43***	−.39***

*Note.* Emotional exhaustion and Depersonalization are scales from the MBI measure used. SECAB-A = Social and Emotional Competence Assessment Battery for Adults.

*** *p* < .001.

### Correlations Between the SECAB-A Scales and Sociodemographic Indicators

Table 7 depicts the correlation values between each of the SECAB-A scales and age and gender. Associations were small, positive, and statistically significant between age and self-regulation and self-awareness. The remaining correlations were extremely small with variables being barely related (as they were below the threshold of .10).

**Table 7.** Intercorrelation Between SECAB-A Scales and Sociodemographic Indicators.

Sociodemographic indicators	Self-regulation	Self-awareness	Conflict management	Positive relationship	Responsible decision making
Age (in years)	.11**	.10**	.01	.05	.07
Gender	-.05	.03	.03	.04	-.04

*Note.* SECAB-A = Social and Emotional Competence Assessment Battery for Adults.

** *p* < .010.

## Discussion

In recent years, the development of SEC targeting adults has gained momentum in the SEL literature (Durlak et al., 2015), namely, through SEL for teachers (Oliveira, Roberto, Pereira, et al., 2021; Schonert-Reichl, 2017). Thus, the development of theoretically grounded and developmentally adjusted measures that adequately assess SEC and its different domains is needed to ensure reliable empirical evidence. Hence, this study aimed to develop and validate a self-report measure of adults’ SEC following the SEL rationale (Durlak et al., 2015; Elias et al., 1997).

The findings support the established hypothesis with regard to the SECAB-A’s factor structure. Thus, the SECAB-A is composed of three independent questionnaires that assess the specific skills across the five key competences of the SEL framework. The *Intrapersonal Competence Questionnaire*, which presents a bidimensional factor structure, assesses the intrapersonal dimensions of SEC through a knowledge dimension (i.e., self-awareness) and a behavioral dimension (i.e., self-regulation), thus confirming H1a. The *Interpersonal Competence Questionnaire* also presents a two-factor structure with both scales integrating knowledge (i.e., social awareness) and behavioral aspects (i.e., relationship skills), sustaining H1b. This questionnaire assesses the interpersonal dimensions of SEC through a dimension focusing on the establishment and maintenance of strong and supportive relations (i.e., positive relationship) and a second dimension addressing the ability to manage conflict when differences and negative interactions emerge within interpersonal relationships (i.e., conflict management). Finally, the *Responsible Decision-Making Competence Questionnaire*, which presents a one-factor model, assesses the competence of responsible decision-making, thus confirming H1c. CFA showed that these factor solution models fit the data better than the alternative factorial solutions tested, providing initial support for the construct validity of the proposed theoretical structure. Results from CFA on the alternative models tested also highlight the complexity of SEC assessment due to the interdependence of different areas of competence (Durlak et al., 2015; Elias et al., 1997). This finding may even help explain the lack of parsimonious instruments to evaluate the five core domains of SEC, reinforcing the parsimony of a three-questionnaire organization of the SECAB-A.

Coefficient omegas supported the adequate internal consistency of the five SECAB-A scales. The correlations between the SECAB-A scales were positive, significant, and moderate to large, suggesting that these dimensions are associated. This finding also acknowledges the difficulty in clearly delimiting boundaries between each specific SEC, as they all intertwine to allow individuals to effectively respond to life challenges (Durlak et al., 2015; Tolan et al., 2016). Analysis of the scores for the test–retest reliability and agreement also adds initial support to the psychometric quality of this measure.

The results provided support for convergent validity, with positive and moderate intercorrelations found between self-awareness and self-regulation and cognitive reappraisal and positive affect, thus sustaining H2. As expected, these results suggest that higher levels of self-awareness and self-regulation are associated with one’s ability to change the emotional impact of a situation and to feel more positive affect (Durlak et al., 2015). Discriminant validity was also corroborated by the findings, with small and nonsignificant intercorrelations found between emotional exhaustion and the interpersonal SEC and responsible decision-making skills (confirming H3a) and between depersonalization and the intrapersonal SEC (sustaining H3b). These results provided initial evidence of the SECAB-A’s convergent and discriminant validities against the external measures.

Finally, intercorrelations between the study measures and the sociodemographic indicators suggested that adults’ perceived own SEC are not associated with their gender. This finding is in line with the thesis that with the growing centrality of SEC in education, gender differences tend to be minimized as SEC is associated with the socialization process and can be learned (Mattingly & Kraiger, 2019; Núñez et al., 2008). Nevertheless, this topic is not consensual in the literature, and further research should be conducted to understand the role of gender, education, and sociocultural contexts in adults’ SEC (e.g., Cabello et al., 2016; S. N. Taylor & Hood, 2011). However, with regard to age, there was a positive, small, and statistically significant association between age and the intrapersonal SEC, suggesting that older adults appear to self-report higher levels of both self-awareness and self-regulation. The same pattern was not found for interpersonal or responsible decision-making competencies.

### Limitations and Future Research

This study presents some limitations that should be taken into consideration in future studies. First, although the secondary school level (equivalent to high school) is mandatory under the Portuguese Education System (young adults aged 18 years or above are required to have at least high-school graduation), a nonprobabilistic online sample with high academic qualifications was considered (most participants had a bachelor or master’s degree). Males were also underrepresented in the sample. The imbalance of sample characteristics might have been motivated by the fact that, due to COVID-19 restrictions, data collection was conducted exclusively online, and in Portugal, females tend to engage more online, and the internet is used more profusely by individuals with higher schooling and socioeconomic levels (ANACOM, 2021; Observador, 2015). Moreover, the data collection software prompted participants to complete missing items and automatically excluded incomplete responses which could also have impacted the results. Therefore, although an online data validation protocol was used, it is important for future research to resort to different data collection methods and validate the SECAB-A factorial structure and its psychometric characteristics with different adult samples, namely, by comparing different levels of schooling, analyzing gender differences, diverse professional groups, and people with lower levels of SEC. Also, due to the sample’s imbalance in terms of gender, age, and academic qualification, it was not possible to test for structural invariance across the groups. Thus, invariance analysis should be tested in future studies. Future research should also analyze and consider whether it is necessary to maintain the modification indices used in this study to provide the best fit to the data as their interpretation, although theoretically supported, may be idiosyncratic to this sample. Also, in terms of sample characteristics, the convenience subsample used represented a significant sample size reduction compared to the total sample, and slight differences regarding the sociodemographic characteristics were found between them. Nevertheless, these sociodemographic differences may be due to the fact that this subsample represents an active population subgroup while the general sample includes college students and retired participants. Although no statistically significant differences were found between the samples regarding the SECAB-A scales, test–retest and validity analyses should be reassessed in future studies with larger and diversified samples (e.g., college students and other professionals). It would also be important for future studies to rely on larger samples and consider context-specific correlational benchmarks (e.g., psychological characteristics–performance) to test for criterion-related validity, namely, predictive.

Furthermore, despite the generally good indicators of test–retest reliability and agreement, Pearson correlation scores suggested higher variability particularly across the Self-awareness, Positive relationship, and Responsible decision-making scales. This variability may be related to the data collection period that coincided with a period of sociocultural instability in Portugal (i.e., the COVID-19 pandemic) that may have contributed to explaining part of this variability (i.e., during the data collection period re-adaptations were necessary since face-to-face work was being reintroduced in Portugal after the first lockdown). Moreover, the stress/well-being cycle that accompanies school terms may have affected teachers’ self-evaluations as the subsample used was composed of elementary school teachers. Therefore, more studies assessing SECAB-A’s test–retest should be conducted. Another limitation is the exclusive use of self-report measures to study SECAB-A’s validity as well as the absence of a convergent validity test for the interpersonal and responsible decision-making competencies, due to the lack of validated, culturally adapted, and translated instruments. Therefore, despite the initial and important validity analysis and the preliminary indicators that have shown to be adequate and in the expected direction, additional criterion-related validity and construct validity research are still needed. Thus, other external measures resorting to observational and/or behavioral measures should be considered to further investigate the validity of SECAB-A, when a period of greater stability is reached and the restrictions to enter the contexts due to COVID-19 are lifted. Finally, despite the endorsed options to directly reduce SDB, relatively high means were obtained on some scales, namely, Self-awareness. Therefore, future research should include a measure for SDB to test and control possible effects of the bias. It may also be important due to possible ceiling effects, which may interfere with research on the intervention, for future studies to test SECAB-A with a 7-point Likert-type response scale, as this length is considered to better identify SDB (Larson, 2019).

### Study Impact

Despite the aforementioned limitations, the findings of this study advance important contributions to both research and practice, thus providing preliminary support for the validity and adequacy of the SECAB-A as a theoretically based measure to assess adults’ social and emotional competencies. The latest recommendations in the SEL literature emphasize the need to develop theoretically and empirically based interventions for the promotion of adults’ own SEC (Durlak et al., 2015). Moreover, across previous research, different measures covering different specific skills within the scope of SEC tend to be used, which increases between-studies heterogeneity and diminishes the reliability of the empirical evidence on SEL contributions (Jennings et al., 2017; Monnier, 2015; Oliveira, Roberto, Pereira, et al., 2021). In this scenario, the SECAB-A may be an important resource for this field of research by presenting an adequate, reliable, and valid theoretically grounded measure that assesses the specific skills within the five core SEC domains in a single and parsimonious measure. On the one hand, for researchers, the SECAB-A may contribute to suppressing the need for valid and developmentally adjusted holistic SEC assessment measures that allow for methodologically robust studies, both in educational settings and within occupational health and business settings where research on SEL is gaining momentum due to its positive impacts on well-being, health, and performance, and decrease in psychological distress (Jennings et al., 2017; Monnier, 2015; Oliveira, Roberto, Pereira, et al., 2021; Oliveira, Roberto, Veiga-Simão, & Marques-Pinto, 2021). On the other hand, this measure may be a useful resource for practitioners, as the evaluation of SEC can provide strong clues for intervention needs, thus contributing to the establishment of intervention goals and contents. Furthermore, by presenting three independent questionnaires that cover all the specific skills within the five core SEC domains, the SECAB-A also enables separate use of the different questionnaires facilitating researchers’ and practitioners’ adaptation of the selected measures, according to the nature of their studies. Moreover, as the SECAB-A is a generic measure for the assessment of adults’ SEC (i.e., it is not a group-specific measure, e.g., for teachers), these research and practical implications can be extended to multiple areas that are now becoming more aware of the need to promote and assess adults’ SEC, namely, in their professional contexts. In view of the specific job demands of the 21st century, “employability skills” (e.g., being able to self-regulate, manage emotions, solve problems, be creative and make decisions, be empathic and demonstrate integrity, develop positive relationships, communicate and collaborate effectively with others; California Career Resource Network, 2021) are receiving growing interest across employers and Human Resources departments, particularly within the scope of social and health services (e.g., teachers, physicians, and nurses) but also within corporate contexts (e.g., white-collar professionals and bank employees) due to the soaring centrality of workers’ performance, occupational health, and work–life balance which can benefit from the training of these competencies (Mattingly & Kraiger, 2019; Monnier, 2015). By being a general measure that covers all the five core domains of SEC with good validity indicators, the SECAB-A also paves the way for different versions which, with slight adaptations to the items’ content, might be adapted to different specific groups, in line with what has occurred with the MBI (Maslach et al.,1996). Finally, as Portuguese is one of the most spoken languages worldwide and the most spoken language in the Southern Hemisphere, SECAB-A can also facilitate, with the necessary adaptations, cross-cultural studies and facilitate the expansion of research and intervention across different adult populations worldwide.
